# Assessing the therapeutic effects of DPP4 Inhibitors on TGF-β2-induced Lens Opacity

**DOI:** 10.1016/j.exer.2026.110967

**Published:** 2026-03-09

**Authors:** Niki Talebian, Pei-Kang Liu, Joanne Lee, Ya-Ling Lin, Dimitrios Stavropoulos, Chi-Yu Chen, Kun-Che Chang

**Affiliations:** a Department of Ophthalmology, Louis J. Fox Center for Vision Restoration, University of Pittsburgh School of Medicine, Pittsburgh, PA, 15219, USA; b Department of Ophthalmology, Kaohsiung Medical University Hospital, School of Medicine, College of Medicine, Kaohsiung Medical University, Kaohsiung, Taiwan; c Department of Neurobiology, Center of Neuroscience, University of Pittsburgh School of Medicine, Pittsburgh, PA, 15213, USA; d Department of Bioengineering, University of Pittsburgh, Pittsburgh, PA, 15213, USA; e Graduate Institute of Medicine, College of Medicine, Kaohsiung Medical University, Kaohsiung, 80708, Taiwan

**Keywords:** DPP4 inhibitor, Sitagliptin, Saxagliptin, Vildagliptin, Cataract, Epithelium-mesenchymal transition, Posterior capsular opacification

## Abstract

Posterior capsular opacification (PCO) is a common complication following cataract surgery, resulting from the epithelial-mesenchymal transition (EMT) of lens epithelial cells (LECs). PCO continues to represent a significant public health challenge due to its detrimental impact on visual outcomes, with many patients requiring additional interventions. In this study, we evaluated the potential efficacy of DPP4 inhibitors—specifically Sitagliptin, Saxagliptin, and Vildagliptin—in mitigating TGF-β-induced EMT in LECs. We hypothesized that these inhibitors could reduce fibrotic changes in LECs and slow the progression of PCO. Human LECs and mouse lenses were pretreated with DPP4 inhibitors prior to exposure to TGF-β2. The expression of EMT markers, including fibronectin, vimentin, and α-smooth muscle actin, was assessed using immunostaining, Western blotting, and RT-qPCR. Both cellular and lens explant cultures demonstrated that DPP4 inhibitors attenuated the expression of TGF-β2-induced EMT markers by blocking Smad2 phosphorylation. Notably, lens explant cultures treated with Vildagliptin showed a reduction in lens opacity induced by TGF-β2. These findings suggest that DPP4 inhibitors, particularly Vildagliptin, may offer a novel, non-invasive pharmacological approach to preventing PCO by targeting TGF-β signaling pathways.

## 1. Introduction

Cataract is one of the leading causes of vision impairment and blindness worldwide (Hashemi et al., 2020; Heruye et al., 2020). A recent study estimated that cataracts account for approximately 40% of global blindness, affecting 17 million people (Vision Loss Expert Group of the Global Burden of Disease et al., 2024). Aging and diabetes are recognized as major risk factors for cataract development (Lim et al., 2020). Given increasing life expectancies and the growing diabetic population, the number of individuals affected by cataract-related blindness is projected to reach 40 million by 2025 (Fang et al., 2022; Vision Loss Expert Group of the Global Burden of Disease et al., 2024).

Currently, cataract surgery is the most effective treatment, typically involving the removal of the opaque lens and its replacement with an artificial intraocular lens to restore vision (Thompson and Lakhani, 2015). Despite the success of cataract surgery, a significant postoperative complication is the development of posterior capsule opacification (PCO), which adversely affects visual outcomes and quality of life (Kubo et al., 2018; Sun et al., 2021). PCO occurs in 20%–50% of adults following cataract surgery (Chang et al., 2017; Sun et al., 2021) presenting a major public health challenge due to its high prevalence and impact on postoperative recovery. Regardless of surgical technique, cataract surgery disrupts the ocular integrity, triggering a wound-healing response that elevates transforming growth factor-beta (TGF-β) levels in the eye (Wallentin et al., 1998; Wormstone et al., 2021). TGF-β induces epithelial-mesenchymal transition (EMT) in residual lens epithelial cells (LECs) by modulating key transcription factors (Hachana and Larrivee, 2022; Kubo et al., 2018). As a result, LECs proliferate and migrate to the posterior capsule rather than differentiate into normal lens fiber cells (Gotoh et al., 2007; Li et al., 2011; Nahomi and Nagaraj, 2018; Nam et al., 2021). This aberrant differentiation of capsular epithelial cells contributes to the onset of PCO (Zhang et al., 2017).

EMT is characterized by a series of molecular and morphological changes, including the activation of specific transcription factors, altered expression of cell surface markers, cytoskeletal reorganization, and the loss of epithelial markers coupled with the gain of mesenchymal markers (Shu and Lovicu, 2017; Kalluri and Weinberg, 2009; Yang et al., 2020). During EMT, LECs lose their characteristic polarity and cell-to-cell adhesion properties, proliferate, migrate, and differentiate into myofibroblasts, which secrete extracellular matrix (ECM) components that contribute to capsular wrinkling and opacity (Chang et al., 2017; Kubo et al., 2018; Lovicu et al., 2016; Wang et al., 2024).

Dipeptidyl peptidase-4 (DPP4), also known as CD26, is a multifunctional serine protease involved in a variety of physiological processes, including immune modulation, fibrosis, and glucose metabolism. DPP4 inactivates key substrates such as glucagon-like peptide-1 (GLP-1) and glucose-dependent insulinotropic polypeptide (GIP), which are critical in regulating glucose homeostasis (Zhang et al., 2021). In addition to its metabolic roles, DPP4 is implicated in cell migration, proliferation, and differentiation in contexts such as cancer and fibrosis (Li et al., 2020). DPP4 inhibitors, including Sitagliptin, Linagliptin, Saxagliptin, and Vildagliptin, were initially developed as antidiabetic agents and are approved by the Food and Drug Administration (FDA) (Ahren, 2007) and the European Medicines Agency (EMA) (Mathieu and Degrande, 2008). These inhibitors prevent the degradation of GLP-1, enhancing insulin secretion and improving blood glucose control without causing significant hypoglycemia or weight gain (Kang et al., 2021; Lim et al., 2015; Yoshida et al., 2023). Beyond their metabolic effects, DPP4 inhibitors also possess pleiotropic properties, including anti-inflammatory, anti-fibrotic, and immunomodulatory actions (Lim et al., 2015; Yoshida et al., 2023). In addition, DPP4 inhibitors were also reported to inhibit the phosphorylation of Smad2 and Smad3, which are transcription factors that are effectors of TGF-β-induced EMT and fibrosis (Yoshida et al., 2023). Linagliptin has also been reported to reduce EMT in various in vitro models, including TGF-β-stimulated human Tenon's fibroblasts (Przekora et al., 2017). In this study, we explored the potential therapeutic effects of DPP4 inhibitors in mitigating TGF-β-induced EMT in human lens epithelial cells (LECs) and mouse lens explants. By blocking EMT-related signaling pathways, DPP4 inhibitors may attenuate the fibrotic response in LECs and thereby reduce the progression of PCO. Our findings suggest that DPP4 inhibitors, particularly Vildagliptin, could offer a promising non-invasive pharmacological strategy to prevent or manage PCO, potentially improving postoperative outcomes in cataract surgery patients.

## 2. Materials and methods

### 2.1. Materials and cell culture

Recombinant human transforming growth factor-beta 2 (TGF-β2, activated form, 302-B2) was obtained from R&D systems (Minneapolis, MN, USA), while DPP4 inhibitors Sitagliptin (SML3205), Saxagliptin (SML3046), and Vildagliptin (SML2302) were obtained from Sigma-Aldrich (Saint Louis, MO, USA). All DPP4 inhibitors were dissolved in DMSO (Sigma-Aldrich, D8418). Fetal human lens epithelial cells (FHL-124, Kindly provided by Dr. Ram H. Nagaraj at University of Colorado) (Raghavan and Nagaraj, 2016) were cultured in Dulbecco's Modified Eagle Medium (DMEM; Gibco, 11885–084) supplemented with 5% fetal bovine serum and 100 units/mL of penicillin/streptomycin (Gibco, 15140122). Cells were cultured in a humidified incubator with 5% CO2 at 37 °C (Wang et al., 2024).

### 2.2. Ex vivo mouse lenses cultivation

This research was conducted in compliance with the Association for Research in Vision and Ophthalmology Statement for the Use of Animals in Ophthalmic and Vision Research. For this study, 8-week-old C57BL/6 wild-type mice of both genders were utilized. The animals were kept in microisolator cages on a 12-h day/night cycle. Mice were euthanized by isoflurane liquid inhalant (Medline, 66794–017-25) and cervical dislocation (IACUC #21018446). Mouse lenses were isolated and cultured in DMEM supplemented with 0.1% bovine serum albumin, and 100 units/mL of penicillin/streptomycin in a humidified incubator with 5% CO2 at 37 °C. To assess the lens opacity, mouse lenses were pretreated with DMSO and DPP4 inhibitors (1 μM) for 1 h, followed by TGF-β2 (1 ng/mL) treatment for 3 days. Lens opacity was evaluated by placing the lenses above a metal grid with a 250 μm spacing interval, and transparency was analyzed under consistent light intensity and exposure using an EVOS M5000 microscope (Invitrogen, Thermo Fisher Scientific). Increased opacity was indicated by the enhanced darkness of the ring within the lens, which blocked light transmission.

### 2.3. MTT assay

An MTT assay was performed to find a concentration of DPP4 inhibitors that would not impair cell viability in the experiments. FHL-124 cells were treated with four different concentrations of DPP4 inhibitors, 0.01 μM, 0.1 μM, 1 μM, and 10 μM, dissolved in DMSO, and the cell viability was analyzed. The confluent cells were treated with the compounds mentioned above for 24 h. After adding the MTT reagent (Sigma-Aldrich, 475989–1 GM), cells were incubated following the manufacturer's protocol. The absorbance was measured to quantify the viability of cells and to determine the highest non-toxic concentration of DPP4 inhibitors to be used in our experiments.

### 2.4. Cell assays

FHL-124 cells were initially plated in 6-well and 24-well plates. At 70% confluency, the cells were pretreated under five different conditions: (1) 1% DMSO with PBS as the baseline control, (2) 1% DMSO followed by TGF-β2 to induce EMT, (3) Sitagliptin pretreatment followed by TGF-β2, (4) Saxagliptin pretreatment followed by TGF-β2, and (5) Vildagliptin pretreatment followed by TGF-β2. The same concentration of DMSO was used for the control groups. The cells were pretreated with DMSO or DPP4 inhibitors for 1 h and then induced for EMT with TGF-β2 (1 ng/mL) or a control vehicle for 24 h before fixation and staining.

### 2.5. Immunofluorescence staining

After 24 h of exposure to TGF-β and DPP4 inhibitors, the FHL-124 cells were fixed with 4% paraformaldehyde (Fisher Scientific, 50–980-488) for 15 min and then washed with phosphate-buffered saline (PBS) three times for 10 min. Cells were blocked by blocking buffer containing 5% Bovine Serum Albumin (Sigma-Aldrich, A4161) and 0.2% Triton X-100 (Sigma-Aldrich, X100) in PBS for 1 h. Cells were incubated with anti-fibronectin antibody (1:500, Abcam, ab2413) overnight at 4 °C. The next day, cells were washed for 10 min three times with PBS and incubated with goat anti-rabbit Alexa Fluor Plus 555 secondary antibody (1:500, Thermo Fisher Scientific, A-11034) and counterstained with DAPI (1:500, Thermo Fisher Scientific, D1306) at the room temperature for 1 h. Fluorescence images were obtained using an EVOS M5000 microscope (Invitrogen, Thermo Fisher Scientific).

### 2.6. Western blot

Cells were collected by RIPA buffer (Thermo Fisher Scientific, 89900) and centrifuged at 15,000×*g* for 15 min at 4 °C to collect the supernatant. To quantify protein concentration, a bicinchoninic acid assay (BCA, Thermo Fisher Scientific, 23225) was performed. Protein samples were subjected to SDS-PAGE (4%–20% Mini-PROTEAN TGX precast protein gels, Bio-Rad, 4561094), followed by transferred to polyvinylidene difluoride (PVDF) membrane (Bio-Rad, 1704272) using the Trans-Blot Turbo^™^ Transfer System (Bio-Rad). After blocked with 5% non-fat dry milk for 1 h, the membranes were incubated overnight at 4 °C with primary antibodies against Fibronectin (1:1000, Abcam, ab2413), Vimentin (1:1000, Abcam, 92547), p-Smad2 (1:1000, Cell Signaling Technology, #), Smad2 (1:1000, Cell Signaling Technology, #) and internal control glyceraldehde 3-phosphate dehydrogenase (GAPDH, 1:1000, Cell Signaling Technology, 2118S). Membranes were washed three times for 15 min each with TBST, followed by incubation with secondary antibody Goat anti-rabbit IgG (H + L) secondary antibody (Invitrogen, 31460) in blocking buffer for 1 h at room temperature with gentle shaking. Protein expression was visualized using Clarity Western ECL substrate (Bio-Rad, 1705061) and imaged with iBright CL750 Imaging System (Invitrogen, Thermo Fisher Scientific). The data were then analyzed and quantified using ImageJ software.

### 2.7. Real-time qPCR (RT-qPCR)

FHL-124 cells were cultured at a density of 1.5 × 10^5^ cells per well in 6-well plates. Cells were pretreated with DPP4 inhibitors (1 μM) for 1 h, and then stimulated with TGF-β2 (1 ng/mL) for 24 h. In a parallel experiment with mouse lenses, the same protocol was followed, with the exception that the TGF-β exposure lasted for 3 days. After treatment, lenses and cells were sonicated and lysed by the RNeasy Plus Mini Kit (Qiagen, 74134). The extracted RNA was reverse-transcribed into complementary DNA (cDNA) using the iScript^™^ cDNA Synthesis Kit (Bio-Rad, 1708891) following the manufacturer's protocol. The cDNA synthesis was performed under the following thermal cycler with the following conditions: 5 min at 25 °C, 20 min at 46 °C, and 1 min at 95 °C, followed by cooling at 4 °C. Quantitative PCR (qPCR) was then conducted using the iTaq^™^ Universal SYBR Green Supermix (Bio-Rad, 1725121) and specific primer sets for human or mouse are listed below: mouse fibronectin 1 (Fn1) forward 5′-CCCTATCTCTGATACCGT TGTCC-3′, reverse 5′-TGCCGCAACTACTGTGATTCGG-3’; mouse alpha smooth muscle actin 2 (Acta2) forward 5′- TGCTGACAGAGGCACCACTGAA −3′, reverse 5′- CAGTTGTACGTCCAGAGGCATAG −3’; mouse vimentin (Vim) forward 5- CGGAAAGTGGAATCCTTGCAGG −3′, reverse 5′- AGCAGTGAGGTCAGGCTTGGAA −3’; mouse GAPDH forward 5’ - CATCACTGCCACCCAGAAGACTG-3′, reverse 5′- ATGCCAGTGAGCTTCCCGTTCAG −3′, human fibronectin 1 (FN1) forward 5′- ACAACACCGAGGTGACTGAGAC −3′, reverse 5′- GGACACAACGATGCTTCCTGAG −3’; human alpha smooth muscle actin 2 (ACTA2) forward 5’-CTATGCCTCTGGACGCACAACT −3′, reverse 5′- CAGATCCAGACGCATGATGGCA −3’; human vimentin (VIM) forward 5- AGGCAAAGCAGGAGTCCACTGA −3′, reverse 5′ -ATCTGGCGTTCCAGGGACTCAT −3’; human GAPDH forward 5′- GTCTCCTCTGACTTCAACAGCG −3′, reverse 5’ -ACCACCCTGTTGCTGTAGCCAA −3’. Reactions were performed using the QuantStudio 3 Real-Time PCR System (Thermo Fisher Scientific). The expression levels of the target genes were normalized to the internal control gene GAPDH. Relative gene expression levels were calculated using the comparative cycle threshold (Ct) method (ΔΔCt), and fold changes in gene expression were compared to the control group treated with DMSO only.

### 2.8. Statistical analysis

Statistical analyses were performed to calculate the significance of the experimental data obtained. All data were represented as mean ± SEM of at least three independent experiments. The fluorescence intensity of immunofluorescence images was quantified by normalizing integrated density to cell number in each group using ImageJ. For Western blot analyses, protein expression levels were normalized using ImageJ to compare band intensities. Comparisons of means among more than two groups were carried out by one-way ANOVA, followed by Tukey's post hoc test for multiple comparisons. Statistical significance was defined as a p-value <0.05. Analyses were performed using GraphPad Prism (version 10, GraphPad Software Inc.).

## 3. Results

### 3.1. DPP4 inhibitors suppress TGF-β2-induced fibronectin expression in FHL-124 cells

**Fig. 1.**
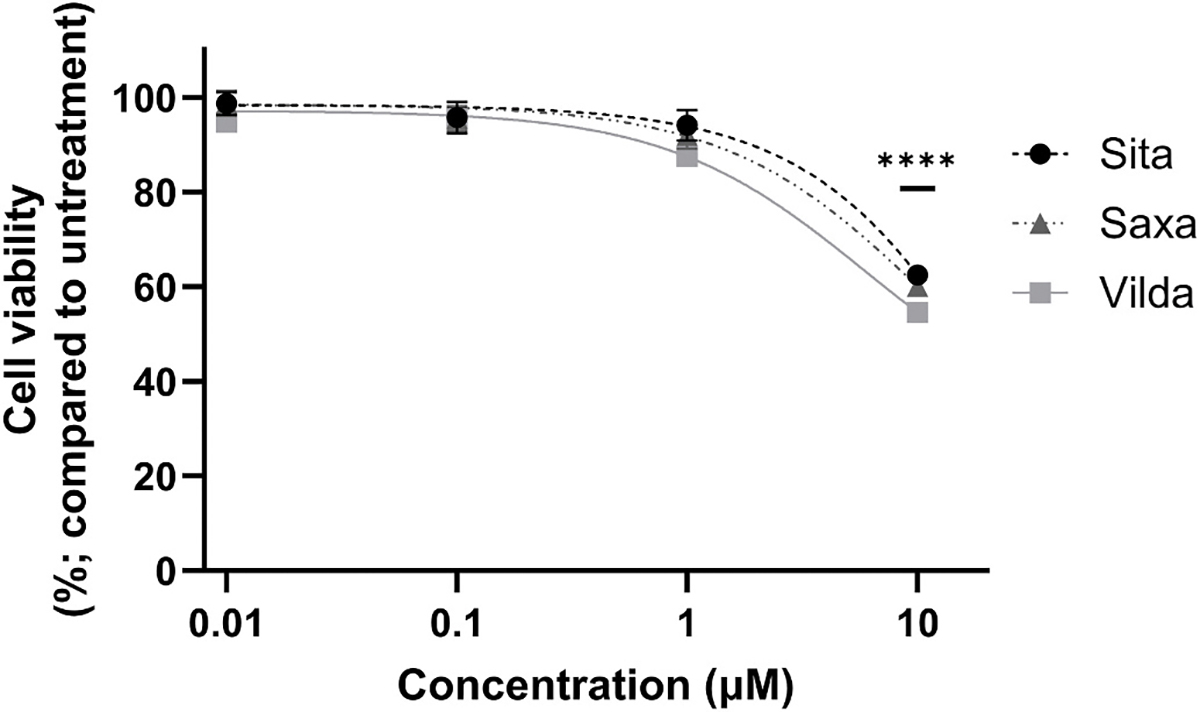
The optimal DPP4 inhibitor concentrations for cell viability in FHL-124 cells were determined by MTT assay. FHL-124 cells were treated with DPP4 inhibitors for 24 h. Cell viability is represented as the percentage compared to untreated cells. Sitagliptin (Sita), Saxagliptin (Saxa), and Vildagliptin (Vilda) are abbreviated as shown. N = 3 independent experiments per group, data were expressed as the mean ± SEM. ****p < 0.0001 by one-way ANOVA and post hoc *t*-test with Tukey correction.

**Fig. 2.**
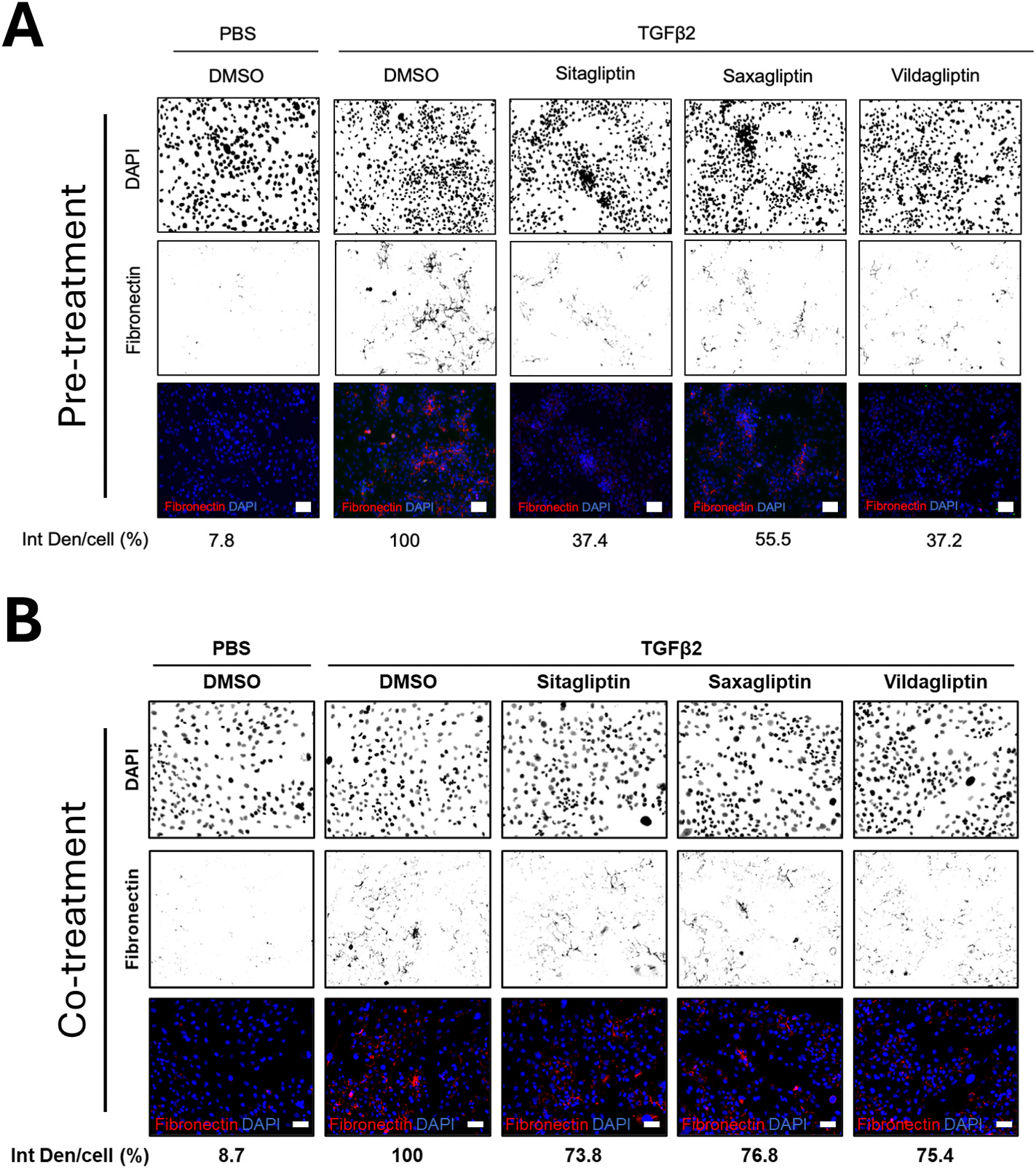
Representative immunofluorescence images stained for fibronectin (red) and DAPI (blue). **(A)** FHL-124 cells were pretreated with DMSO or 1 μM of each DPP4 inhibitor for 1 h followed 1 ng/mL TGF-β2 stimulation for 24 h. **(B)** FHL-124 cells were treated with 1 ng/mL TGF-β2 in the presence of either 1 μM of each DPP4 inhibitor or DMSO for 24 h. Int Den = integrated density. Int Den/cell was quantified and normalized to DMSO + TGF-β2 treatment (set to 100%). Scale bar = 100 μm. (For interpretation of the references to colour in this figure legend, the reader is referred to the Web version of this article.)

To determine whether DPP4 inhibitors have the potential to suppress epithelial-mesenchymal transition (EMT) in FHL-124 cells, we first established their optimal dosage to avoid cytotoxic effects. Based on MTT assay results, a concentration of 1 μM was identified as appropriate, as it maintained cell viability in FHL-124 cells (Fig. 1). Immunofluorescence staining was then employed to assess the expression of fibronectin (Fn1) in FHL-124 cells undergoing TGF-β2-induced EMT with DPP4 inhibitor pretreatment. The immunofluorescence images revealed minimal fibronectin expression in the control group (DMSO + PBS), while the TGF-β2 group exhibited a marked increase in fibronectin expression (Fig. 2A). This confirmed that TGF-β2 induces EMT, leading to enhanced fibronectin production and fibrotic activity in these cells. Notably, pretreatment with Sitagliptin, Saxagliptin, or Vildagliptin significantly reduced fibronectin expression compared to the TGF-β2 group without DPP4 inhibitors (Fig. 2A). Moreover, we compared the efficacy of DPP4 inhibitors under different treatment timings. While simultaneous administration of TGF-β2 and DPP4 inhibitors also resulted in a reduction of fibronectin expression, the inhibitory effect was more pronounced in the pretreatment group (Fig. 2A and B).

These findings demonstrated a significant increase in fibronectin expression in the DMSO + TGF-β2 group compared to the DMSO + PBS group. However, pretreatment with DPP4 inhibitors (Sitagliptin, Saxagliptin, and Vildagliptin) effectively reduced fibronectin levels, aligning with the qualitative observation of diminished red fluorescence. Furthermore, our data suggest that early intervention with DPP4 inhibitors provides superior protection against the initiation of the EMT activation.

### 3.2. DPP4 inhibitors suppress TGF-β2-induced protein expression of EMT markers in FHL-124 cells

**Fig. 3.**
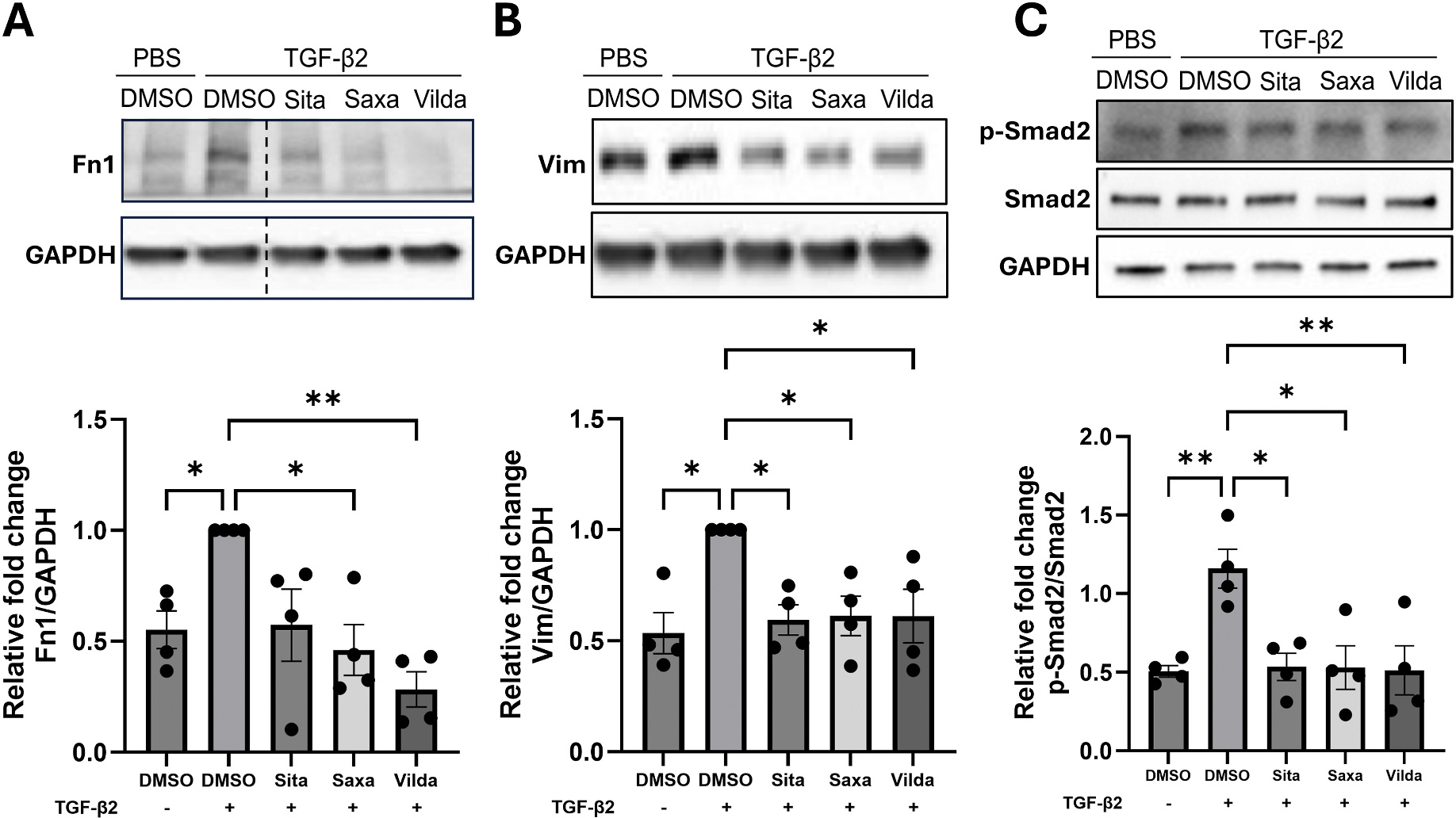
Typical Western blotting shows the protein levels of **(A)** Fn1 and **(B)** Vim and the quantification of images in FHL-124 cells treated with TGF-β (1 ng/mL) for 24 h following DPP4 inhibitors 1 h pretreatment (Sitagliptin, Saxagliptin, Vildagliptin; 1 μM). **(C)** Immunoblotting analysis of Smad2 and p-Smad2 levels in FHL-124 cells treated with DPP4 for 1 h followed by TGF-β2 (1 ng/mL) treatment for 24 h. The dashed line in the fibronectin blot represents a cut in the membrane image, indicating that this was a single membrane containing all samples, with only the relevant section shown here for clarity. Protein and gene expressions were normalized to GAPDH. Sitagliptin (Sita), Saxagliptin (Saxa), and Vildagliptin (Vilda) are abbreviated as shown. N = 4 independent experiments per group, data were expressed as the mean ± SEM. *p < 0.05, **p < 0.01 by one-way ANOVA and post hoc *t*-test with Tukey correction.

Following the immunofluorescence analysis of Fn1, the expression of EMT markers vimentin (Vim) and fibronectin (Fn1) were further examined at the protein level using Western blot analysis. FHL-124 cells were pretreated with the DPP4 inhibitors Sitagliptin, Saxagliptin, and Vildagliptin before exposure to TGF-β2. The results demonstrated that treatment with DMSO followed by TGF-β2 significantly increased the protein expression of Fn1 (Fig. 3A) and Vim (Fig. 3B) compared to the PBS + DMSO control group. Notably, the TGF-β2-induced upregulation of Fn1 and Vim was markedly suppressed in cells pretreated with DPP4 inhibitors, indicating their efficacy in mitigating EMT-related protein expression.

### 3.3. DPP4 inhibitors suppress EMT activation by inhibiting TGF-β2-induced Smad2 phosphorylation in FHL-124 cells

The Smad2 signaling pathway is important to EMT activation. To further confirm the inhibitory mechanisms of DPP4 inhibitors, the relative abundance of p-Smad2 was assessed by immunoblotting. FHL-124 cells were pretreated with DPP4 inhibitors and then exposed to TGF-β2. Following TGF-β2 stimulation, the expression level of p-Smad2 increased in FHL-124 cells. However, pretreatment with DPP4 inhibitors effectively reduced this induction by TGF-β2 (Fig. 3C).

### 3.4. DPP4 inhibitors suppress TGF-β2-induced mRNA expression of EMT markers in FHL-124 cells

**Fig. 4.**
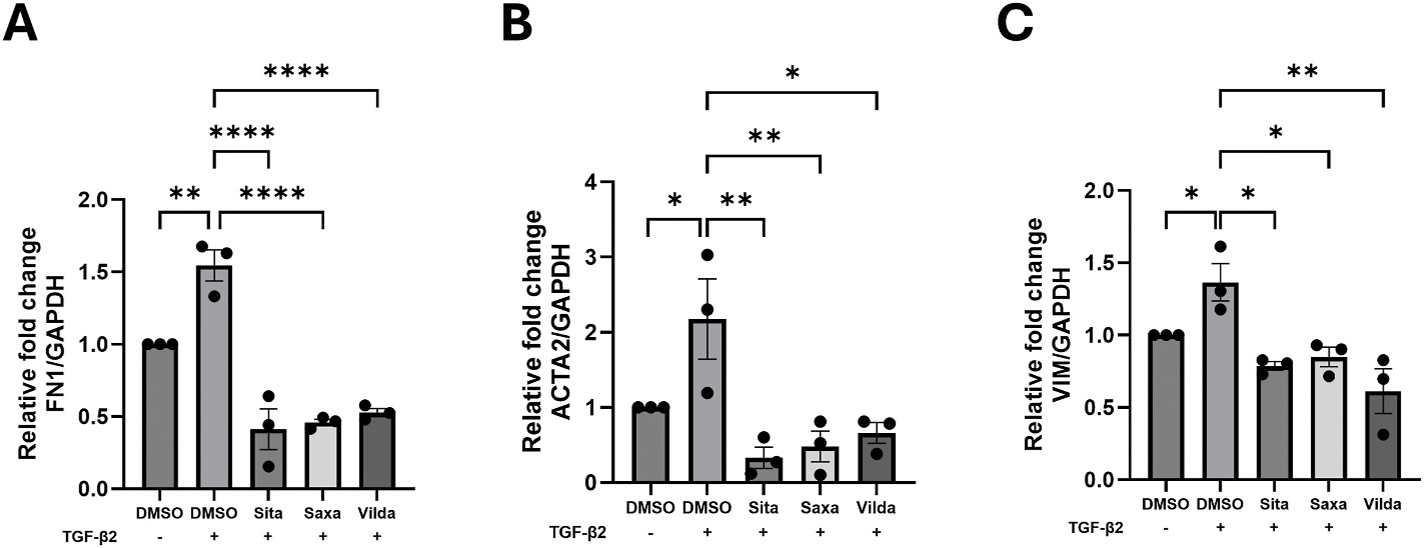
Expression levels of **(A)** FN1, **(B)** ACTA2, and **(C)** VIM in FHL-124 cells were assessed by RT-qPCR. Cells pretreated with 1 μM DPP4 inhibitors (Sitagliptin, Saxagliptin, or Vildagliptin) for 1 h followed by TGF-β2 (1 ng/mL) treatment for 24 h. Relative fold changes are represented as 2^−ΔΔCt^ compared to the DMSO only group. Sitagliptin (Sita), Saxagliptin (Saxa), and Vildagliptin (Vilda) are abbreviated as shown. Gene and protein expressions were normalized to GAPDH. N = 3 independent experiments per group, data were expressed as the mean ± SEM. *p < 0.05, **p < 0.01, and ****p < 0.0001 by one-way ANOVA and post hoc *t*-test with Tukey correction.

The expression of EMT markers, including Fibronectin 1 (FN1), alpha-smooth muscle actin 2 (ACTA2), and Vimentin (VIM), was analyzed using RT-qPCR in FHL-124 cells pretreated with DPP4 inhibitors followed by exposure to TGF-β2. FN1 expression was significantly elevated in the DMSO + TGF-β2 treatment group compared to the DMSO + PBS control group. However, pretreatment with Sitagliptin, Saxagliptin, and Vildagliptin resulted in a significant reduction in the expression levels of FN1 (Fig. 4A), ACTA2 (Fig. 4B), and VIM (Fig. 4C) compared to the TGF-β2 group, highlighting the inhibitory effect of DPP4 inhibitors on EMT marker expression.

### 3.5. DPP4 inhibitors alleviate TGF-β2-induced opacity in mouse lenses and reduce EMT markers expression in primary mouse lens epithelial cells

**Fig. 5.**
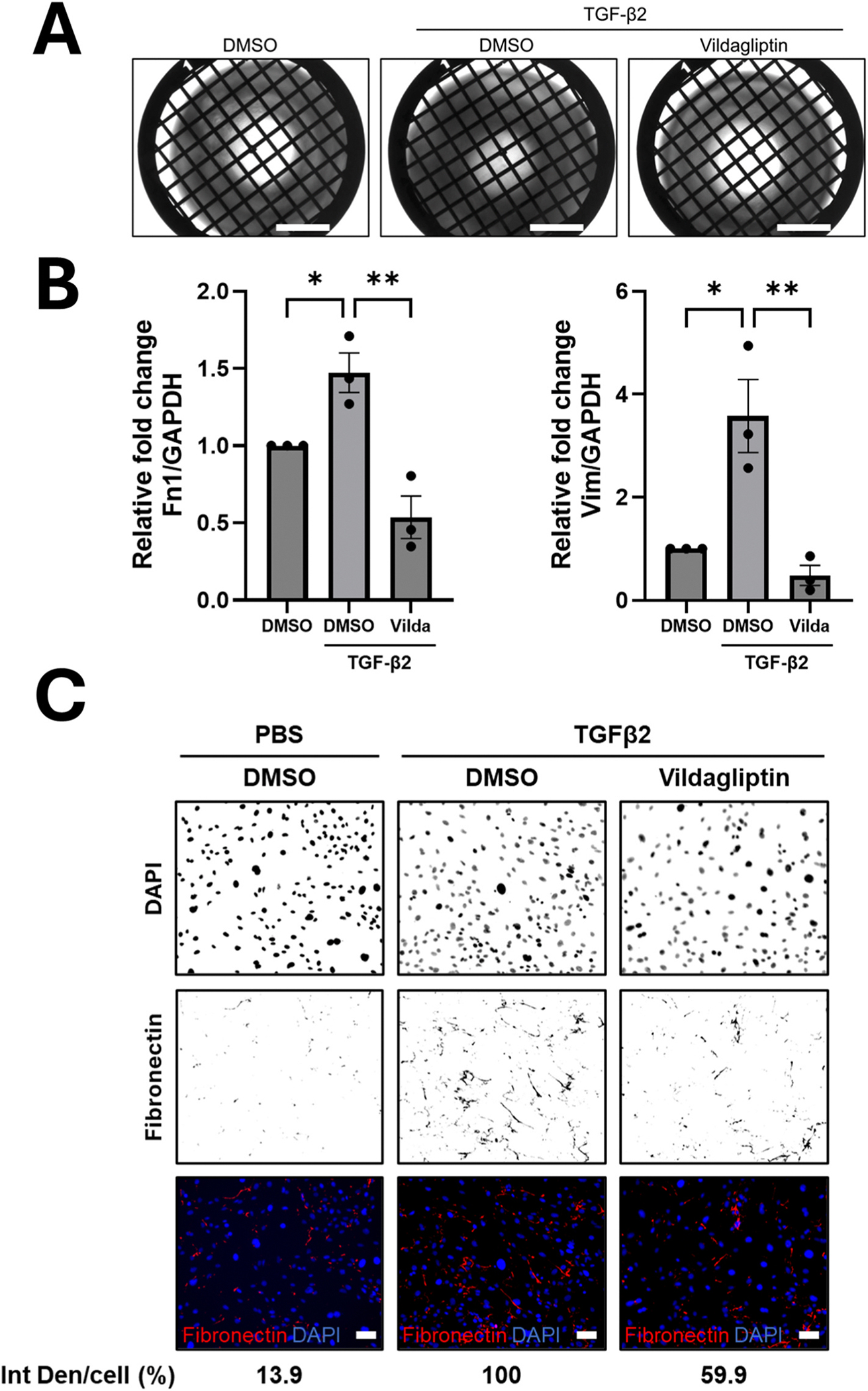
Effect of Vildagliptin on TGF-β2-induced lens opacity and EMT marker expression in mouse lenses and primary mouse lens epithelial cells. **(A)** A significant difference in the appearance of the lenses treated with DMSO, TGFβ2 (1 ng/mL), and TGFβ2 with Vildagliptin. Scale bar = 300 μm. **(B)** Treatment with 1 ng/mL TGFβ2 in lenses elevated the expression levels of EMT markers, fibronectin 1 (Fn1), and vimentin (Vim). Pretreatment with Vildagliptin for 1 h alleviated the TGFβ2-induced EMT marker elevation. Relative fold changes are represented as 2^−ΔΔCT^ compared to DMSO only group. N = 3 independent experiments per group, data were expressed as the mean ± SEM. *p < 0.05, **p < 0.01 by one-way ANOVA and post hoc *t*-test with Tukey correction. Representative immunofluorescence images stained for fibronectin (red) and DAPI (blue). **(C)** Mouse primary lens epithelial cells were pretreated with DMSO or 1 μM Vildagliptin for 1 h followed 1 ng/mL TGF-β2 stimulation for 24 h. Int Den = integrated density. Int Den/cell was quantified and normalized to DMSO + TGF-β2 treatment (set to 100%). Scale bar = 100 μm. (For interpretation of the references to colour in this figure legend, the reader is referred to the Web version of this article.)

The ability of DPP4 inhibitors to mitigate TGF-β2-induced EMT was assessed using an explanted mouse lens model. Lenses were pretreated with Vildagliptin or DMSO (vehicle) before exposure to TGF-β2 or PBS (vehicle) for three days. In the images, greater opacity (indicating reduced light transmission) appears as darker regions. Control lenses exhibited minimal opacity, consistent with in vitro findings. In contrast, TGF-β2 treatment induced pronounced opacity, whereas lenses pretreated with Vildagliptin showed substantially less opacity following TGF-β2 induction (Fig. 5A). The expression of EMT markers Fn1 and Vim in the lenses was evaluated via RT-qPCR. Both markers were significantly elevated in the DMSO + TGF-β2 group compared to controls, while Vildagliptin pretreatment markedly reduced their expression (Fig. 5B).

To further validate these findings in a relevant cell model, we conducted a mouse primary lens epithelial cell culture (Yu et al., 2024). Immunofluorescence staining was performed to evaluate the expression of fibronectin. Consistent with our *ex-vivo* observations, the DMSO + TGF-β2 group exhibited a significant increase in fibronectin expression compared to the PBS + DMSO. Notably, this upregulation was suppressed with Vildagliptin pretreatment (Fig. 5C). Collectively, these results from both whole-lens explants and primary cell cultures demonstrate that Vildagliptin attenuates TGF-β2-induced EMT and the subsequently reduces lens opacity.

## 4. Discussion

PCO is a significant challenge in ophthalmology, where Nd:YAG laser capsulotomy is the standard treatment. However, laser treatment carries risks such as macular edema, retinal detachment, and increased intraocular pressure. Therefore, the relevance of non-invasive methods is raised. In this study, we focused on the role of TGF-β2 in inducing EMT and its potential contribution to fibrotic changes in PCO. TGF-β2 mainly induces EMT through the Smad pathway, which plays a part in fibrosis development (Chen et al., 2021). DPP4 inhibitors were evaluated as potential pharmacological interventions to inhibit the pathway that could be applied for the prevention of EMT-driven fibrosis and subsequent PCO. A previous study (Lovicu et al., 2004) reported that TGF-β typically induces localized focal fibrotic plaques in the lens. The specific morphological manifestation often depends on the growth factor concentration and the duration of exposure. In our study, treatment with 1 ng/mL of TGF-β2 for 3 days resulted in a more circumferential opacification pattern. Despite these morphological variations, the underlying pathological process remains consistent, driven by the aberrant accumulation of extracellular matrix proteins such as FN1 which subsequently leads to the loss of crystalline organization. This is further substantiated by our molecular data, which demonstrates a direct correlation between the upregulation of EMT markers and the observed optical changes in the mouse lens. Among the DPP4 inhibitors studied, Sitagliptin, Saxagliptin, and Vildagliptin showed significant suppression of EMT markers: Vim, Fn1, and Acta2, indicating their strong anti-fibrotic potential. These findings align with previous studies, including one investigating the role of DPP4 inhibitors in glaucoma (Yoshida et al., 2023). These inhibitors also attenuated TGF-β2-mediated Smad2 phosphorylation, a critical step in EMT induction via the TGF-β2/Smad2 pathway (Valcourt et al., 2005). The reduction in p-Smad2 levels aligns with the suppression of downstream EMT markers, suggesting that DPP4 inhibitors directly regulated the TGF-β2/Smad2 signaling to mitigate fibrosis. DPP4 inhibitors attenuate TGF-β signaling through several mechanisms, including the disruption of TGF-β receptor complex formation (Ohm et al., 2023; Shi et al., 2015) and the inhibition of Smad signaling (Yoshida et al., 2023). Previous studies have shown that soluble DPP4 can directly bind to the TGF-β receptor (Huang et al., 2023) or activate Smad signaling via PAR2 (Lee et al., 2020) in renal epithelial cells. Consistent with these findings, we propose that DPP4 inhibitors may competitively interfere with the interaction between TGF-β and its receptor complex or disrupt DPP4-mediated receptor sensitization. Such interference would subsequently attenuate Smad2 phosphorylation and suppress the downstream EMT activation, even in the presence of exogenous active TGF-β2. This evidence supports the broader applicability of DPP4 inhibitors as anti-fibrotic agents in ocular diseases. Our data demonstrated that DPP4 inhibitors modulate TGF-β2 signaling to suppress EMT markers, supporting their potential as a pharmacological approach for the management of PCO. In addition, blocking the EMT molecular pathways by DPP4 inhibitor also offers other therapeutic strategies for EMT-related ocular diseases such as Age-related macular degeneration (AMD) (Liu et al., 2023) and proliferative vitreoretinopathy (PVR) (Chen et al., 2015). Moreover, Diabetes significantly increased the incidence of PCO after 18 months compared to the non-diabetic patients (Hayashi et al., 2002; Praveen et al., 2014). DPP4 inhibitors are believed to provide additional benefits for diabetic patients with PCO.

Since only a small panel of DPP4 inhibitors was explored in the present study, expanding investigations to include other clinically available inhibitors would provide a more comprehensive understanding of their anti-fibrotic efficacy in ocular tissues. Post-treating TGF-induced EMT is challenging, as TGF-Smad2 signaling occurs, making it difficult to rescue any effects without prior intervention. Therefore, in this study, DPP4 inhibitors were considered for pretreatment as a preventive approach. Combinatorial treatments with other pharmacological agents can enhance EMT and fibrosis inhibition. Given the interplay between TGF-β2 and several other signaling pathways such as ERK and mTOR, and the complexity of this pathway, future studies on the molecular effect of DPP4 inhibitors on these pathways are warranted to fully explain the anti-fibrotic role of DPP4 inhibitors. Another limitation of our study is the use of *in-vitro* and *ex-vivo* models but the lack of *in-vivo* models. Therefore, future research should include animal models to enable mechanistic studies and assess the direct and long-term effects of DPP4 inhibitors on lens capsule clarity and visual outcomes.

**Fig. 6.**
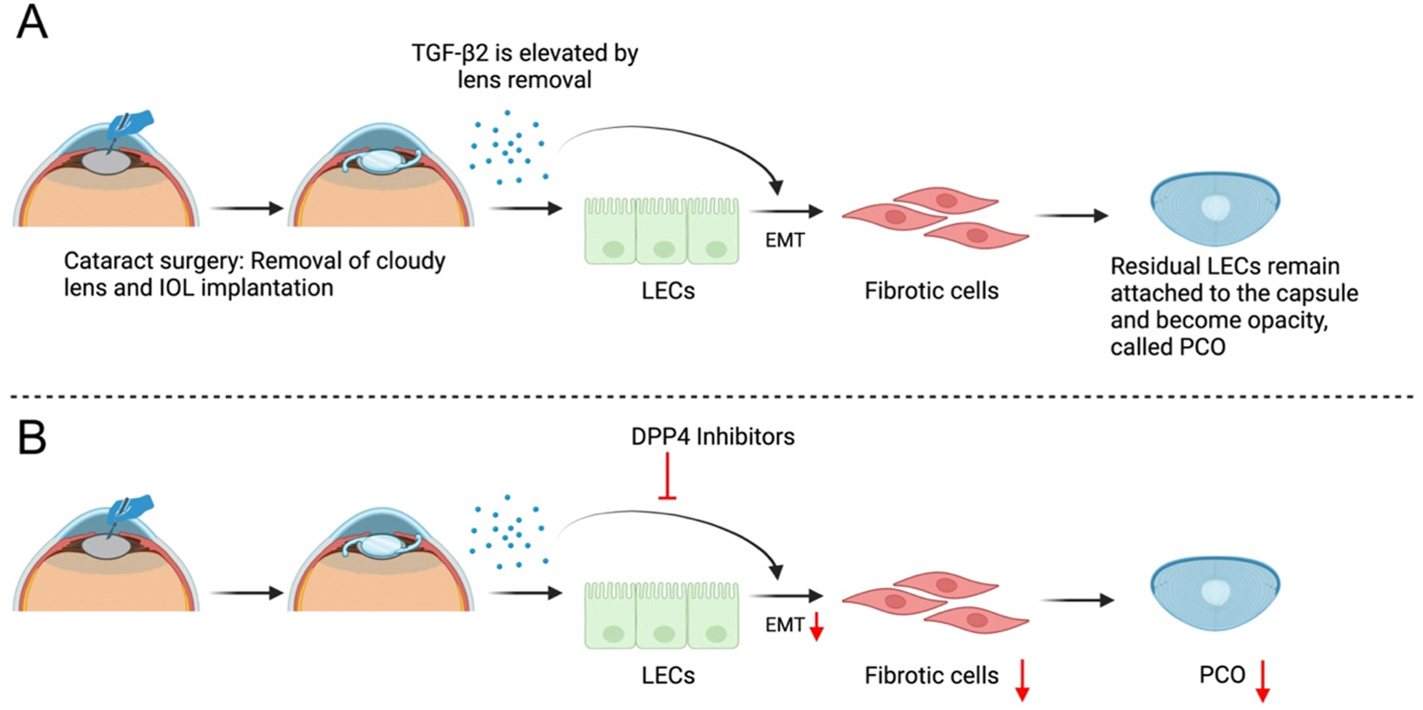
Schematic diagram of the inhibitory effects of DPP4 inhibitors involved in PCO progression. **(A)** TGF-β2 is elevated in the eye after lens removal and causes epithelial-mesenchymal transition (EMT) on lens epithelial cells (LECs) in the capsule, leading to posterior capsular opacification (PCO). **(B)** DPP4 inhibitor treatment attenuates TGF-β2-induced EMT and alleviates PCO progression.

In summary, the current study shows that exposure to TGF-β2 induces EMT and lens opacity (Fig. 6A). DPP4 inhibitors have the potential to block TGF-β2-induced EMT in lens epithelial cells, offering a promising new, non-invasive pharmacological approach to prevent PCO following cataract surgery (Fig. 6B). Sitagliptin, Saxagliptin, and Vildagliptin all demonstrated significant anti-EMT effects, indicating their clinical potential as safer and less complication-prone alternatives to current treatments. Moreover, as highlighted by this study, DPP4 inhibitors could extend beyond their traditional role in diabetes management and open new therapeutic avenues for addressing fibrotic changes in various tissues.

## 5. Conclusions

Given PCO significantly affects a large proportion of individuals who have undergone cataract surgery, its management represents a priority task concerning public health. If the efficacy of DPP4 inhibitors is confirmed in follow-up studies, these agents can be administered either topically or locally to help reduce the risk for post-surgical complications following cataract surgery, thus improving the clinical and visual outcomes for cataract patients. PCO is a complex trait, therefore future studies exploring the environmental and genetic factors conferring susceptibility to this condition may help determine at-high-risk patients who can be selectively targeted with DPP4 inhibitors, as well as reveal other biological mechanisms and related inhibitors that can also be targeted for the management of PCO.

## Data Availability

Data will be made available on request.
